# Applied IrO_2_ Buffer Layer as a Great Promoter on Ti-Doping V_2_O_5_ Electrode to Enhance Electrochromic Device Properties

**DOI:** 10.3390/ma15155179

**Published:** 2022-07-26

**Authors:** Tien-Fu Ko, Po-Wen Chen, Kuan-Ming Li, Hong-Tsu Young

**Affiliations:** 1 Department of Mechanical Engineering, National Taiwan University, Taipei City 10617, Taiwan; chahlesko@iner.gov.tw (T.-F.K.); hyoung@ntu.edu.tw (H.-T.Y.); 2 Division of Physics, Institute of Nuclear Energy Research, Taoyuan City 32546, Taiwan

**Keywords:** iridium oxide (IrO_2_), buffer layer, electrochromic device (ECD), cathodic arc plasma (CAP), coloration efficiency (CE)

## Abstract

Electrochromic devices (ECDs) are a promising material for smart windows that are capable of transmittance variation. However, ECDs are still too expensive to achieve a wide market reach. Reducing fabrication cost remains a challenge. In this study, we inserted an IrO_2_ buffer layer on Ti-doped V_2_O_5_ (Ti:V_2_O_5_) as a counter electrode using various Ar/O_2_ gas flow ratios (1/2, 1/2.5, 1/3 and 1/3.5) in the fabrication process. The buffered-ECD resulted in a larger cyclic voltammetry (CV) area and the best surface average roughness (Ra = 3.91 nm) to promote electrochromic performance. It was fabricated using the low-cost, fast deposition process of vacuum cathodic arc plasma (CAP). This study investigates the influence of the IrO_2_ buffer/Ti:V_2_O_5_ electrode on ECD electrochemical and optical properties, in terms of color efficiency (CE) and cycle durability. The buffered ECD (glass/ITO/WO_3_/liquid electrolyte/IrO_2_ buffer/Ti:V_2_O_5_/ITO/glass) demonstrated excellent optical transmittance modulation; ∆T = 57% (from T_bleaching_ (67%) to T_coloring_ (10%)) at 633 nm, which was higher than without the buffer (ITO/WO_3_/liquid electrolyte/Ti:V_2_O_5_/ITO) (∆T = 36%). In addition, by means of an IrO_2_ buffer, the ECD exhibited high coloration efficiency of 96.1 cm^2^/mC and good durability, which decayed by only 2% after 1000 cycles.

## 1. Introduction

As part of the drive to mitigate the global energy crisis, one approach is the development of energy saving devices. Electrochromic devices (ECDs) are regarded as excellent candidates for research, as they feature low power usage, reversible changing, low-power drives large optical modulation, and good memory [1,2,3,4]. ECDs are attracting considerable attention since they have practical applications in smart windows for buildings and in heat-insulating glass for airplanes [5,6].

Smart windows are based on electrochromic materials that block solar and indoor sunlight and heat, enabling a reduction in the energy required for air conditioning [7]. A wide variety of ECD materials have been studied, including transition metal oxides, such as nickel oxide (NiO), tungsten oxide (WO_3_), zinc oxide (ZnO), and vanadium oxide (V_2_O_5_) [8,9,10,11].

WO_3_ is a well-known cathodic material and NiO is a common anodic material [12,13,14]. The main drawbacks of the NiO ECDs are the low contrast in optical transmittance and short durability [15,16]. Hence, V_2_O_5_ film has recently been studied as an anodic electrode in ECD, where it can participate in reversible oxidation and reduction reactions, which facilitate the movement of Li ions into/out of the interface electrodes and electrolyte [17,18]. The typical redox reaction of V_2_O_5_ with perchlorate (LiClO_4_) electrolyte can be written as follows [19]:

V_2_O_5_ + xLi^+^ + x^−^ = Li_x_V_2_O_5_ (1)

Many studies have reported that V_2_O_5_ with appropriate dopants, such as Mn, Ag, Al, and Ti [20,21,22,23], can improve the microstructure electrochemical characteristics. The effect of Li^+^ reversibility on Ti-doped V_2_O_5_ (Ti:V_2_O_5_) is particularly interesting [24] and Kumar et al. indicate that the proper ratio of Ti added to V_2_O_5_ could increase the charge density as well as promote the cycling stability [23]. Here, we report an approach that inserts an IrO_2_ buffer layer on the Ti:V_2_O_5_ counter electrode to significantly enhance the high coloration efficiency and optical modulation contrast. The superior performance is ascribed to the buffer layer, which is beneficial for the electrode used in forming ECDs, as it results in prominent durability. The findings show that the buffer layer can enhance the properties of ECDs.

In general, ECD electrode films comprise an anode and a cathode and can be manufactured by several methods, including sputtering [25,26,27], chemical deposition [28,29], sol–gel [30,31], dip coating [32,33], pulsed-laser deposition [34], and electrodeposition [35]. Sputtering is the most common technology for electrode film preparation. In this work, the Ti:V_2_O_5_ and WO_3_ electrodes with the IrO_2_ buffer layer were fabricated using cathodic arc plasma (CAP). This technique has the advantages of high-density plasma that significantly promotes deposition rate, low cost, good film thickness control, a growing porous structure to benefit electrochromism, and is capable of achieving substrate deposition over a larger area without using complicated machinery. However, the CAP technique is not widely applied owing to poorer macroparticle production. This is caused by the effect of the plasma–liquid pool on cathode spots, attaching to the electrode film and making the performance inferior. The harmful macroparticles are the main reason why the CAP technique is not suitable for industrial application. Therefore, we developed a method that made use of Thornton deposition [36,37] to reduce macroparticle size and adjust the process parameters for high-pressure work to produce a self-organized structure, with a high horizontal magnetic field to improve the quality.

In this study, we inserted a buffer into the ECD structure between the liquid electrolyte and the Ti:V_2_O_5_ counter electrode. The structure is a multiple layer sandwich consisting of a pair of transparent conducting oxides (TCOs), a working electrochromic electrode (WO_3_), and a counter electrode (Ti:V_2_O_5_) in a perchlorate (LiClO_4_/PC) liquid electrolyte solution. Throughout the entire study, we found that the IrO_2_ buffer layer was indispensable for the electrochemical properties and optical contrast of ECDs. Buffered-ECDs were comparable to without-Buffered ECDs that were well-equipped with durability through prolonged long cycles. We have developed the CAP technique as an alternative method to deposit film, due to its high deposition rate, low processing costs, and ability to coat a large area substrate. The above process advantages are beneficial for the application of ECDs in green window construction. Furthermore, our goal is to make affordable ECD products. Recently, our research group, P.W. Chen et al. [7], reported a method to fabricate complementary electrochromic devices on WO_3_/NO electrodes, and discussed the influence of different WO_3_ film thicknesses on ECDs.

**Figure 1:**
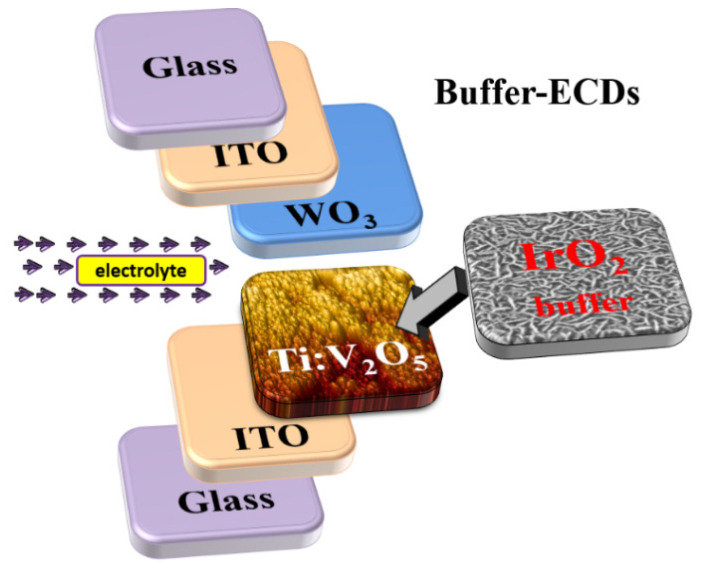
The schematic structure of buffered ECDs.

The elaboration of an IrO_2_ buffer layer on various Ar/O_2_ gas flow ratio parameters of the Ti:V_2_O_5_ counter electrode by the CAP technique is presented. This buffer is deposited on a good interfacial Ti:V_2_O_5_ electrode film based on the optimized Ar/O_2_ gas flow ratio that exhibited the best roughness of amorphous structure and cycle voltammetry (CV), providing a larger enveloped area with sufficient movement of Li ions in/out of the electrode. The configurations of the buffer ECD are illustrated in Figure 1. We investigated the effects of inserting an IrO_2_ buffer layer between a Ti:V_2_O_5_ electrode and an electrolyte, to gain insight into the impact of the buffer on the ECD. Our intent in this approach led to notable mechanical behavior, making the IrO_2_ buffer a potential candidate material for improving ECDs. The influence of an IrO_2_ buffer layer, based on the optimal CV measurements, optical transmittance, and surface roughness, on the Ti:V_2_O_5_ electrode film on the material crystallinity, surface morphology, coloration efficiency, and cycle durability are investigated in this work. We determined that the buffer is a very important layer in the ECD and serves many functions.

## 2. Materials and Methods

### 2.1. Deposition of Ti:V_2_O_5_ Counter Electrode and IrO_2_ Buffer Layer

**Figure 2:**
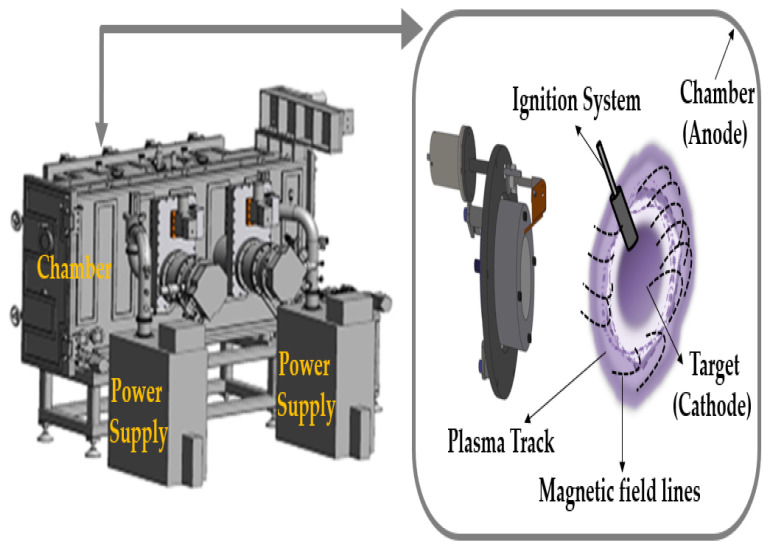
Illustration of cathodic arc plasma (CAP) technique.

The Ti:V_2_O_5_ electrode and IrO_2_ buffer layer were fabricated by the CAP technique showing in Figure 2 and using the targets of Ti-doped V_2_O_5_ (Ti:V = 1:2; 99.95% purity) and metallic iridium (Ir) target (99.95% purity), respectively. We deposited the Ti:V_2_O_5_ films (samples 1–4) using a series of reaction Ar/O_2_ gas flow ratios (1/2, 1/2.5, 1/3, and 1/3.5) as the counter electrode on indium tin oxide (ITO) glass. The IrO_2_ film (sample 5) as the buffer layer with an Ar/O_2_ gas flow ratio of 1/3 was stacked on the Ti:V_2_O_5_ electrode. The deposition parameters implemented are detailed in Table 1.

### 2.2. Preparation of WO_3_ Working Electrochromic Electrode, Transparent and Electrolyte Layer

WO_3_ working electrodes were produced by an identical CAP method using a metallic tungsten (W) target (99.95% purity) and depositing at fixed Ar/O_2_ gas flow ratio of 1/3 on indium tin oxide (ITO) glass substrate (10 × 10 cm^2^) with resistance of 6 Ω/cm^2^. Cathodic arcs can be used for the reactive deposition of various nitrides and oxides. This can largely be overcome by steering the arc rapidly across the surface of the cathode under high working pressure to reduce the spot residence time and limit the formation of erosion craters [7,38]. In recent years, researchers have shifted the emphasis from monolithic coatings to higher performing multilayers and nanocomposites. In those papers, the proposed arc gun setup relies on the flow of argon (for insertion) and oxygen (reaction) to control the formation of the electrode structure.

During the process, each ITO-coated glass sample prior to deposition was washed with deionized water for 2 min to remove surface-bound particles. For the preparation of the UV-curable poly(methyl methacrylate) (PMMA-based polymer electrolyte, 6.00 g of PMMA monomer) and 0.2 M of LiClO_4_ were dissolved in 10.00 g of PC purged by N_2_ gas. Afterward, 0.55 g (5 wt%) of IRGACURE 184 and 0.44 g (4 wt%) of phenothiazine were added. The mixture was prepared in a dark vial to avoid exposure to light and was stirred for 12 h period at 60 °C. The multilayer of the ECDs structure was stacked as follows: ITO (300 nm)/Ti:V_2_O_5_ (80 nm)/IrO_2_ (20 nm)/LiClO_4_PC (100 µm)/WO_3_ (200 nm)/ITO (300 nm), which comprised a working electrode (WO_3_ film on ITO substrate), a counter-electrode (with or without buffer/Ti:V_2_O_5_/ITO films) in 0.2 M LiClO_4_/PC solution. The processes are detailed in Table 2.

### 2.3. Experimental Details

Scanning of the electrochemical characteristics employed cycle voltammetry (CV) and chronoamperometry (CA) (model PGSTAT30, Autolab, Utrecht, The Netherlands) in a three-electrode system with Ti:V_2_O_5_/ITO/glass and IrO_2_ buffer/Ti:V_2_O_5_/ITO/glass as the working electrode, platinum mesh as the counter electrode, and Ag/AgCl as the reference electrode. The film’s optical tansmittance in a wavelength range from 300 nm to 900 nm was measured by an ultraviolet–visible (UV-Vis) spectrophotometer (model DH-2000-BAL, Ocean Optics, Dunedin, FL, USA) based on colored/bleached states. The interface roughness topography was collected by atomic force emission (AFM) (model Innova, Bruker, Billerica, MA, USA). The surface and cross-sectional morphology were obtained from scanning emission microscopy (SEM) (Model S4800, Hitachi, Tokyo, Japan) applied at 15 kV. The crystallographic structure was examined by high-resolution X-ray diffractometer (HRXRD, Model D8, Bruker, Billerica, MA, USA) using CuKα (λ = 0.154 nm) as target with a scan region of 20° to 90°. Raman spectra were collected by 488 nm laser with an average of 100 scans and a power excitation of 45 mW (Model MOF-iHR550, Horiba, Kyoto, Japan).

## 3. Results

### 3.1. Ti:V_2_O_5_/ITO Films: Electrochemical and Optical Properties

**Figure 3:**
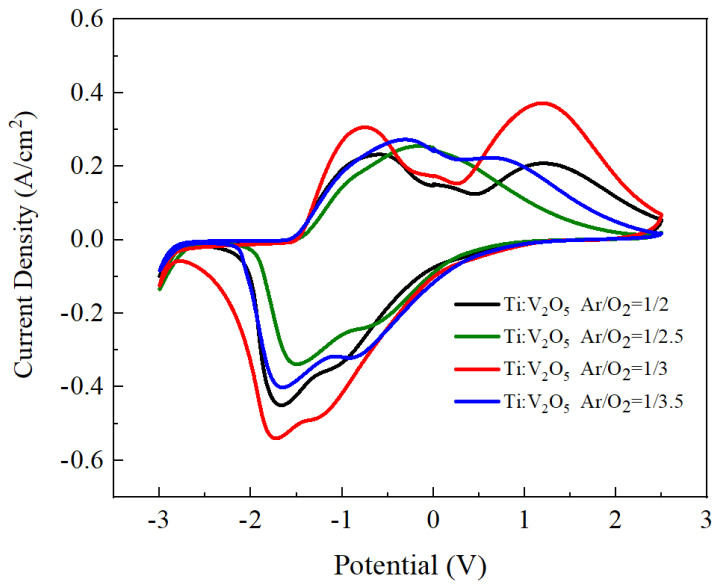
CV curve of Ti:V_2_O_5_ electrode films with various Ar/O_2_ gas flow ratios at the 25th cycle with a scan rate of 100 mV/s.

Cyclic voltammetry (CV) was implemented to acquire the electrochemical properties of the Ti:V_2_O_5_ counter electrode on ITO glass. Figure 3 demonstrates the plot of current density versus potential voltage at the 25th cycle with an applied sweep voltage ranging from −3 V (coloring) to 2.5 V rate (bleaching) and a scan rate of 100 mV/s. The Ti:V_2_O_5_ films were processed with various Ar/O_2_ gas flow ratios (1/2, 1/2.5, 1/3, and 1/3.5) on ITO glass. However, the Ar/O_2_ gas flow ratio of 1/3 resulted a larger enveloped area, indicating that more Li^+^ ions participated in the electrochemical redox reaction [39]. In addition, the CV curve of the Ti:V_2_O_5_ films presents two remarkable cathodic reduction peaks (−1.2 V, −1.7 V) and two anodic peaks (−0.75 V, 1.5 V), which were attributed to Li^+^ intercalaltion and Li^+^ extraction, respectively. This higher optical transmittance modulation is a result of the larger CV envelope area on account of the increased number of Li ions into/out of the electrode interface.

**Figure 4:**
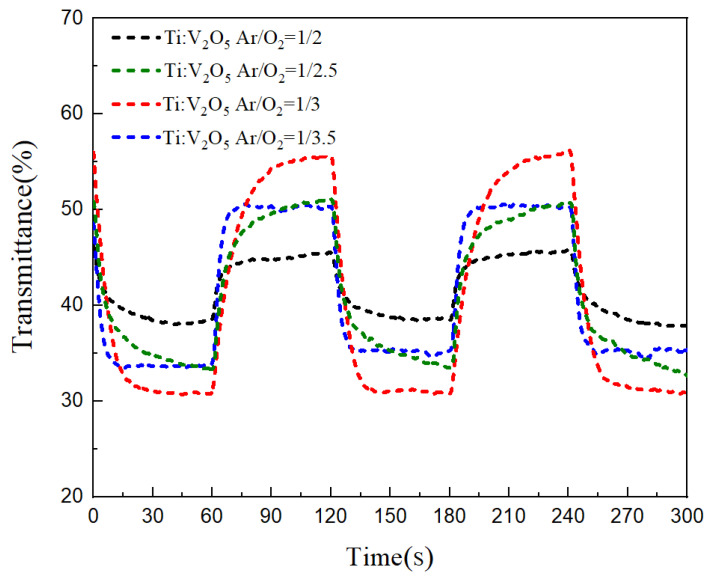
In situ optical transmittance response at 633 nm of Ti:V_2_O_5_ electrode films with various Ar/O_2_ gas flow ratios.

Figure 4 demonstrates the coloring/bleaching in situ optical transmittance of the Ti:V_2_O_5_ electrode films at a wavelength of 633 nm, when applying the same voltage range (−3 V to 2.5 V) with various Ar/O_2_ gas flow ratios.

**Figure 5:**
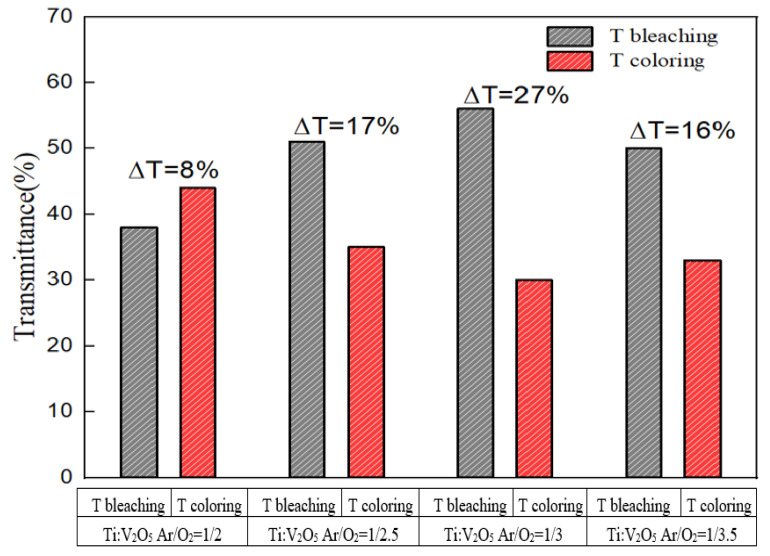
The difference in coloring/bleaching transmittance modulation of Ti:V_2_O_5_ electrode films.

Figure 5 contrasts the coloring/bleaching transmittance modulation. An Ar/O_2_ gas flow ratio of 1/3 of Ti:V_2_O_5_ resulted in the highest optical transmittance of ∆T = 27% (from T_bleaching_ (57%) to T_coloring_ (30%)). This result is in accordance with the cyclic voltammetry (CV) of the 1/3 ratio in Figure 3 and in our previous work [38]. This behavior could be explained by the larger enveloped area and large optical modulation achieved during CV. Electrochromism of the film upon Li^+^ intercalation/extraction was examined by transmittance measurement during the CV process. Considering the highest transmittance (∆T = 27%), we picked an Ar/O_2_ gas flow ratio of 1/3 as the best condition for buffer deposition on the ECD counter electrode.

### 3.2. Ti:V_2_O_5_/ITO Films: Surface Roughness Properties

To understand the interfacial structure, the surface roughness of Ti:V_2_O_5_ counter electrodes on ITO glass was collected by AFM to investigate the film surface topography deposited with different Ar/O_2_ gas flow ratios (samples 1–4). Figure 5 shows the Ti:V_2_O_5_ film visualization of AFM in two dimensions (2D) and three dimensions (3D). In order to present the images, a scan area of 1 × 1 µm^2^ was used. The measurements of topographical parameters are listed in Table 3.

**Figure 6:**
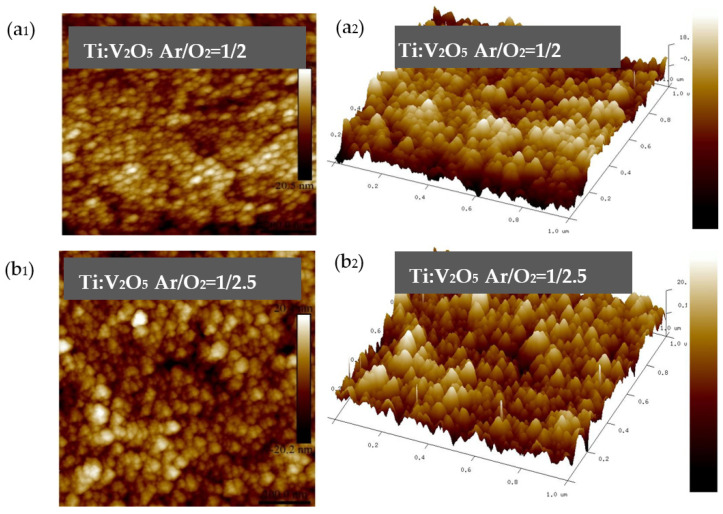

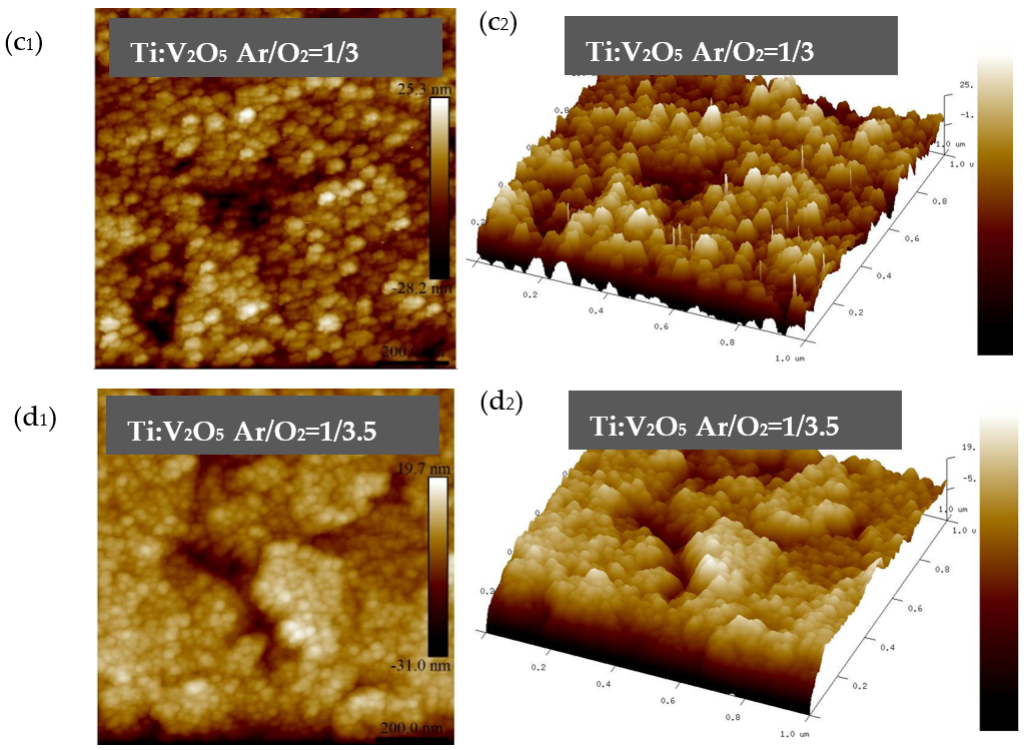
(**a1**–**d1**) 2D AFM visualization of Ti:V_2_O_5_ film; (**a2**–**d2**) 3D AFM visualization of Ti:V_2_O_5_ film.

This work focuses on fabricating Ti:V_2_O_5_ films as a counter electrochromic layer by CAP deposition through four different oxygen and argon ratios. Here, we focus on how to fabricate nanostructure and surface roughness, so as to enhance fast ion insertion/extraction. According to the Thornton model [7,40], a loose-packed porous structure is formed under high-pressure environments. Arc discharge can be operated in high working pressure environments; interesting phenomena may occur such as self-organized synthesis of nanostructure and reduction in macroparticle size. When the arc is operated at higher working pressures, intense ion bombardment and ohmic heating likely allow a switch to thermionic mode; in this case, the erosion craters become smaller, facilitating the formation of a columnar structure. Figure 6 shows that Ar/O_2_ gas flow ratio of 1/3 led to the smallest average roughness (Ra) 3.9 nm and the Ti:V_2_O_5_ film surface was peak-shaped. In contrast with other ratios, the higher (1/3.5) or lower (1/2 and 1/2.5) Ar/O_2_ gas flow ratios led to a higher average roughness (Ra). This result indicated that the smallest Ra was consistent with the cyclic voltammetry (CV) results for the Ar/O_2_ gas flow ratio of 1/3 (sample 3), due to it having a larger enveloped area, which enables the particles to gain energy to migrate along the surface. The enveloped area of the CV curve correlated with the film surface topography. Under the electrochemical redox reaction of the Ti:V_2_O_5_ film, the topography may have the crucial influence on the insertion/extraction of Li ions at the counter electrode.

The surface roughness of the electrode is an essential indicator for light scattering. As shown in Figure 6, the gas flow ratio of 1/3 for Ti:V_2_O_5_ resulted in the highest optical transmittance (∆T = 27%), based on the smallest average roughness (Ra). The higher transmittance obtained might be ascribed to the reduction in light scattering [40]. The counter electrode surface deposited on the smallest Ra value could provide a prominent platform to insert the IrO_2_ buffer layer for fabricating an ideal ECD.

### 3.3. IrO_2_ Buffer/Ti:V_2_O_5_/ITO Films: Coloration Efficiency Analysis

Coloration efficiency (CE) is an imperative criterion for evaluating the assessment of electrochemical performance. It is defined as the change in optical density (ΔOD) per unit insert charge Q_in_ (Q_in_ = Q/A, which is the charge (Q) per unit area (A) of the electrode film). The calculation can be formulated as follows:

CE = ΔOD/Q_in_ (2)

ΔOD = ln (T_bleaching_/T_coloring_) (3)

where T_bleaching_ and T_coloring_ are the transmittance values of the bleaching and coloring states at the specific wavelength of 633 nm, respectively.

**Figure 7:**
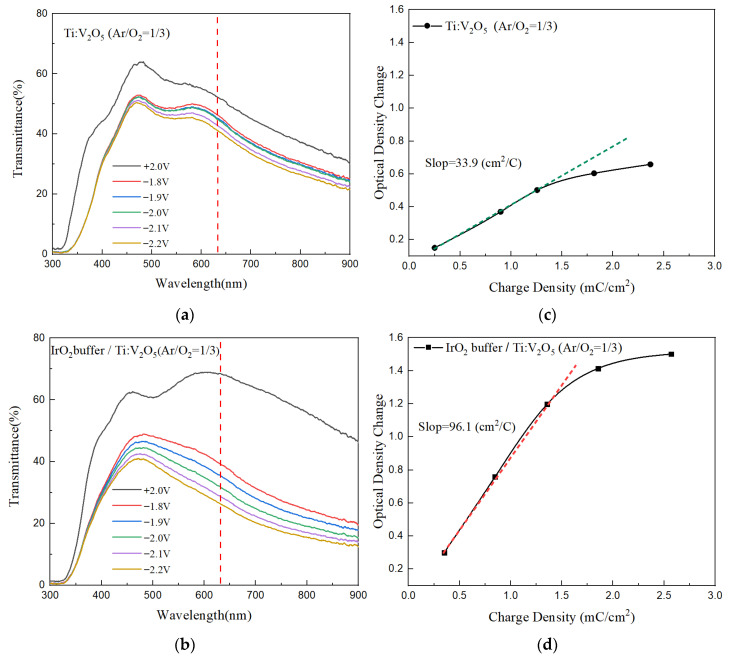
(**a**,**b**) Optical transmittance spectra of Ti:V_2_O_5_ films and IrO_2_ buffer/Ti:V_2_O_5_ films at different applied potential voltages; (**c**,**d**) optical density change vs. charge density of Ti:V_2_O_5_ films and IrO_2_ buffer/Ti:V_2_O_5_ films.

The optical transmittance spectra and CE value of Ti:V_2_O_5_ film and IrO_2_ buffer/Ti:V_2_O_5_/ITO film were determined for the gas flow ratio of 1/3 (sample 3).The amount of charge intercalated (Q_in_) when a negative potential is applied were was integrated by chronoamperometry (CA) measurement. A high CE value (cm^2^C^−1^) is achieved when a large change in optical density is driven by a low amount of inserted charge. In Figure 7a,b, the optical transmittance spectra of Ti:V_2_O_5_ film and IrO_2_ buffer/Ti:V_2_O_5_ film with gradually applied successive potential voltages from +2.0 V (bleaching state) to −2.2 V (coloring state) during a pulsed time interval of 90 s are shown. The change in optical transmittance corresponds to the voltage between the lowest transmittance in the coloring state (T_coloring_) and the highest transmittance in the bleaching state (T_bleaching_). The two electrode films were subjected to gradually decreasing voltages in accordance with the decreasing transmittance. The ΔOD value was nearly linear. The coloring/bleaching states were determined by the positive/negative voltage. IrO_2_ buffer/Ti:V_2_O_5_ film demonstrated lower transmittance than the Ti:V_2_O_5_ film at the coloring state due to inserting a buffer.

Figure 7c,d shows the optical density change vs. charge density of Ti:V_2_O_5_ films and IrO_2_ buffer/Ti:V_2_O_5_ films. The higher CE values indicate that electrodes can provide large optical modulation with small quantities of Li^+^ intercalation/extraction [40,41]. The CE value is calculated from the slope of the line fitted to the linear region of the curve. Compared with the two curves, the CE value of the IrO_2_/Ti:V_2_O_5_ film (96.1 cm^2^/C) is significantly higher than that of the Ti:V_2_O_5_ film (33.1 cm^2^/C). The Ti:V_2_O_5_ film with an IrO_2_ buffer layer provided a higher CE value and is good for promoting optical transmittance.

### 3.4. IrO_2_ Buffer/Ti:V_2_O_5_/ITO Films: X-ray Diffraction and Raman Spectroscopy

**Figure 8:**
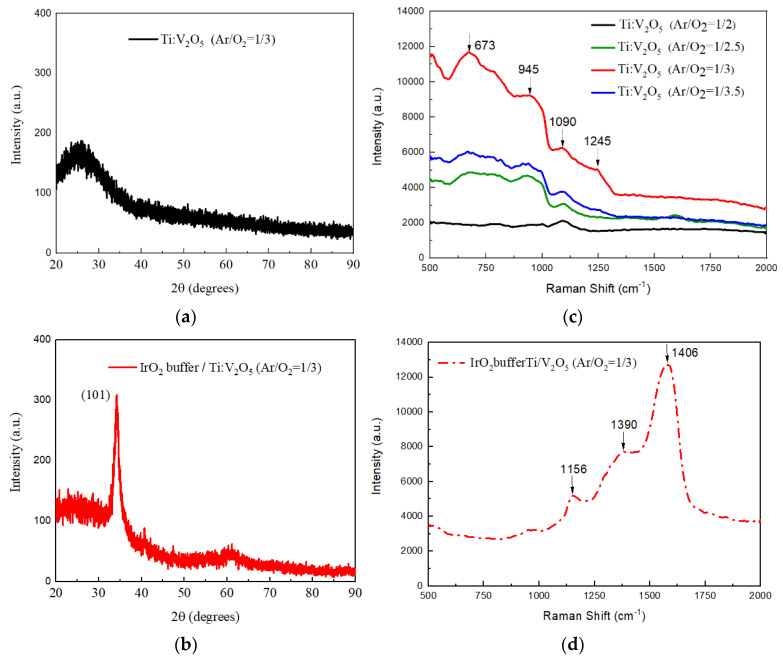
(**a**,**b**) X-ray diffraction of Ti:V_2_O_5_ films and IrO_2_ buffer/Ti:V_2_O_5_ films at an Ar/O_2_ ratio of 1/3; (**c**,**d**) Raman spectroscopy of Ti:V_2_O_5_ films with various Ar/O_2_ ratios and IrO_2_ buffer/Ti:V_2_O_5_ at an Ar/O_2_ ratio of 1/3.

Figure 8a,b shows X-ray diffraction (XRD) of Ti:V_2_O_5_ films and IrO_2_ buffer/Ti:V_2_O_5_ film at an Ar/O_2_ ratio of 1/3. X-ray diffraction was used to assess the crystalline structure and phase in the scanning range from 20° to 90°. To determine the standard pattern identification, use was made of the Joint Committee on Powder Diffraction Standard (JCDPS).

Figure 8a shows that the XRD spectrum of Ti:V_2_O_5_ films (Ar/O_2_ ratio of 1/3) lacked the main peak, which indicates that the film could be an amorphous structure. The characteristics of the Ti:V_2_O_5_ films were not found in the XRD pattern.

Figure 8b shows the XRD spectrum of the IrO_2_ buffer/Ti:V_2_O_5_ film. The most intense diffraction peak was located at a 2θ angle of 34° (JCPDS card no. 47-1049) and corresponded to a preferential orientation of (101), which stacked along the c-axis. As our report investigates the influence of inserting the buffer, it can be observed that the use of IrO_2_ as a buffer on the Ti:V_2_O_5_ film (Ar/O_2_ ratio of 1/3) is an effective way to improve the crystallinity. As shown in Figure 8b, the IrO_2_ buffer/Ti:V_2_O_5_ film can obtain higher optical transmittance ∆T (from T_bleaching_ to T_coloring_) than the Ti:V_2_O_5_ film (Figure 8a). The crystallinity characteristic of the counter electrode films is closely associated with their transmittance.

The vibrations measured in Raman spectroscopy are determined by material structure shown in Figure 8c. Raman spectra of the Ti:V_2_O_5_ films produced by various Ar/O_2_ gas flow ratios (1/2, 1/2.5, 1/3 and 1/3.5) in the wavelength range of 500 cm^−1^ to 2000 cm^−1^ are shown. We found that, with an Ar/O_2_ gas flow ratio of 1/3, the Ti:V_2_O_5_ film exhibits a clearer and more intense band at 673 cm^−1^ and broader bands at 945 cm^−1^, 1090 cm^−1^, and 1245 cm^−1^ compared to other ratios, which have uncharacteristic Raman spectra. However, in comparison with Figure 8d, the IrO_2_ buffer/Ti:V_2_O_5_ film (Ar/O_2_ gas flow ratio of 1/3) has a distinguishable sharp band at 1406 cm^−1^ and broad bands at 1390 cm^−1^ and 1156 cm^−1^, corresponding to the crystalline preferential orientation of (101) in Figure 8b. After inserting the buffer in the Ti:V_2_O_5_ films (Ar/O_2_ gas flow ratio of 1/3), these bands disappeared.

### 3.5. IrO_2_ Buffer/Ti:V_2_O_5_/ITO Films: Surface Morphology

**Figure 9:**
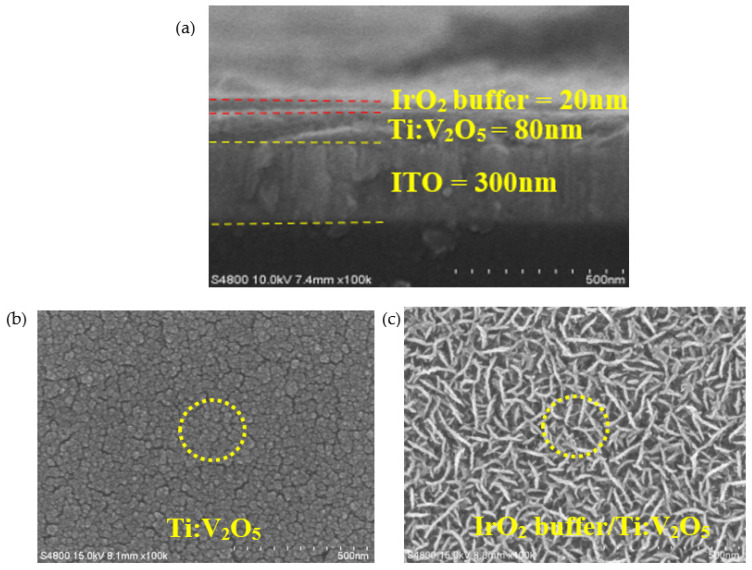
SEM images of surface morphology: (**a**) cross-section morphology of the IrO_2_ buffer/Ti:V_2_O_5_ film at Ar/O_2_ ratio 1/3; (**b**) Ti:V_2_O_5_ film at Ar/O_2_ ratio 1/3; (**c**) IrO_2_ buffer/Ti:V_2_O_5_ film at Ar/O_2_ gas flow ratio of 1/3.

Figure 9a shows the cross-section morphology of the IrO_2_ buffer/Ti:V_2_O_5_ film at an Ar/O_2_ gas flow ratio of 1/3. The 100 nm electrode thickness includes IrO_2_ buffer of 20 nm and Ti:V_2_O_5_ film of 80 nm. The IrO_2_ buffer/Ti:V_2_O_5_ is stacked on indium tin oxide (ITO) glass substrate with a thickness of 300 nm. Figure 9b,c compares the surface morphology of the two materials. Figure 9b shows a Ti:V_2_O_5_/ITO film at an Ar/O_2_ gas flow ratio of 1/3. This is a featureless structure with a smooth and uneven, flakey surface. It might be related to the amorphous structure in Figure 8a imaged by XRD spectroscopy.

In Figure 9c, the IrO_2_ buffer on the Ti:V_2_O_5_ film at an Ar/O_2_ ratio of 1/3 is shown. It has a filamentary and interconnected structure. The morphological result is identical to our previous research [38]. As we found previously, this type of structure is useful for the transfer of Li ions in/out of the interface of the electrode due to the morphology, which possesses a larger inner pore that furnishes a great contact area and abundant porosity. This also proved that the filamentary and interconnected type of structure exhibits higher optical transmittance in Figure 7b and CE value (96.1 cm^2^/C) in Figure 7d of the IrO_2_ buffer/Ti:V_2_O_5_ film than optical transmittance in Figure 7a and CE value (33.9 cm^2^/C) in Figure 7b of the Ti:V_2_O_5_ film.

### 3.6. Buffer ECD: Optical Transmittance and Durability

**Figure 10:**
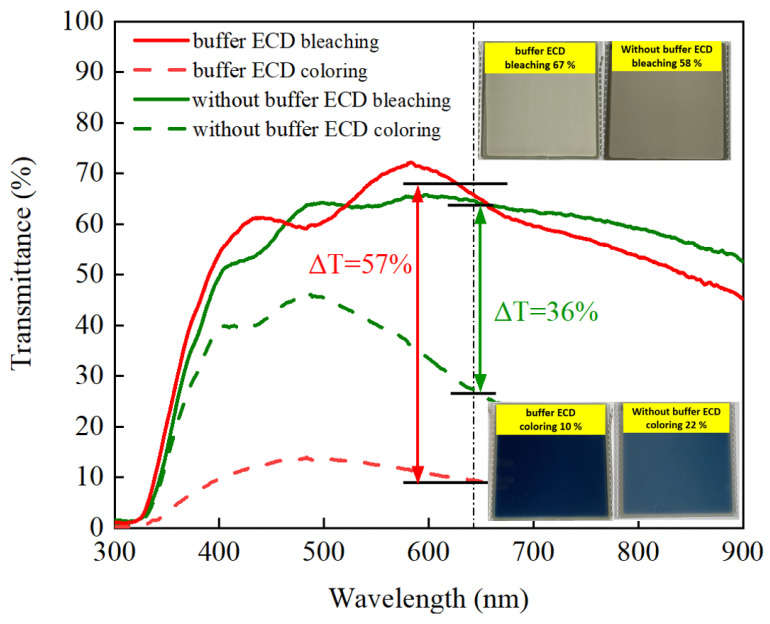
The optical transmittance spectra and image comparison with buffered ECD and ECD without buffer in bleaching/coloring states.

**Figure 11:**
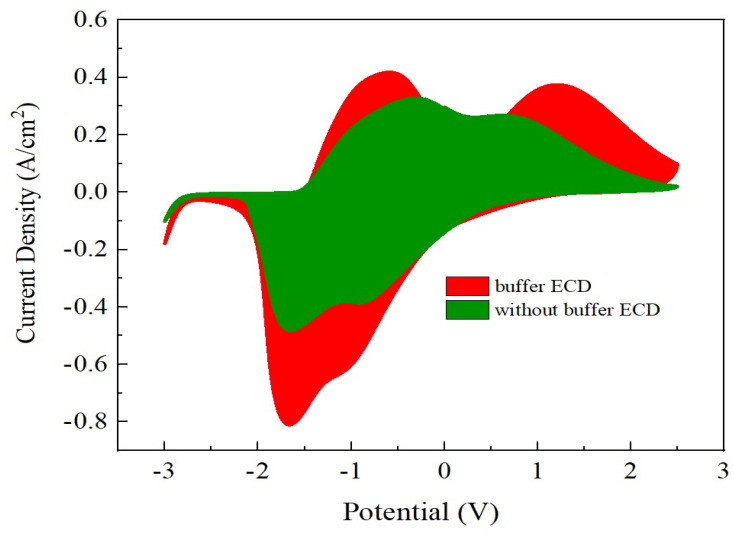
The CV curve comparison between buffered ECD and ECD without buffer.

Figure 10 demonstrates the optical transmittance spectra comparison with buffered ECD and without buffered ECD at bleaching/coloring state, based on the continuous potential voltage 2.5 V to −3 V. For the buffered ECD (glass/ITO/WO_3_/liquid electrolyte/IrO_2_ buffer/Ti:V_2_O_5_(Ar/O_2_ ratio 1/3)/ITO/glass), the optical transmittance modulation was ∆T = 57% (from T_bleaching_ (67%) to T_coloring_ (10%)) at 633 nm, in the range from 300 nm to 1000 nm, which was higher than without the buffer (ITO/WO_3_/liquid electrolyte/Ti:V_2_O_5_ (Ar/O_2_ ratio 1/3)/ITO) (∆T = 36% (from T_bleaching_ (58%) to T_coloring_ (22%)). This could be attributed to a larger CV enveloped area for the buffered-ECD, as shown in Figure 11, on account of the film with greater Li^+^ intercalation/extraction as shown by optical transmittance during the CV analysis. Remarkably, this result was in good agreement with Figure 3, in which the largest CV enveloped area demonstrated the highest transmittance.

**Figure 12:**
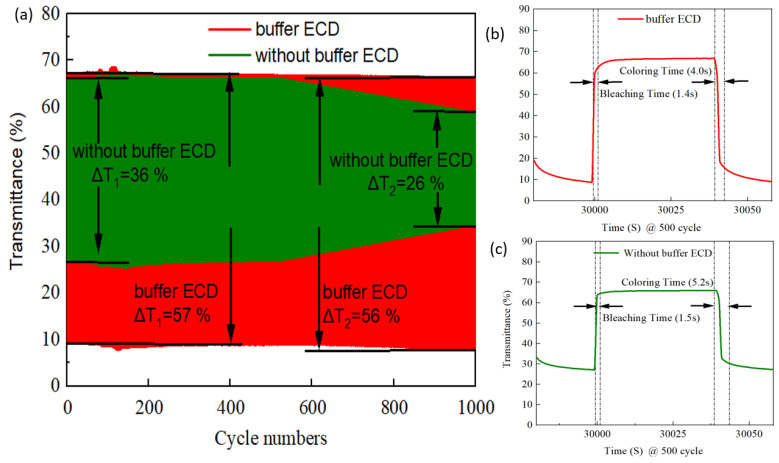
(**a**) The 1000-cycle durability comparison with buffered ECD and ECD without buffer, (**b**) buffered ECD and (**c**) ECD without buffer: switch response time @ 500 cycles.

The durability of the ECD is an important factor to determine whether the product can work in real life. Figure 12a presents the long-life (1000 cycles) durability comparison between the buffered ECD and the ECD without buffer. After 1000 cycles of bleaching/coloring, the buffered ECD had notable durability, retaining 98% (i.e., 2% decay) of the initial value, greater than 78% (22% decay) for the ECD without buffer. The high-contrast optical performance and good durability of the ECD could be attributed to the inserted IrO_2_ buffer on the electrode. We found that both optical transmittance and durability were, in terms of electrode surface morphology, equipped with filamentary and interconnect type structure. The buffer ECD with the IrO_2_ buffer/Ti:V_2_O_5_ electrode provided a higher CE value (96.1 cm^2^/C) to support the Li^+^ intercalation/extraction process. Figure 12b,c illustrates the buffered ECD and the ECD without buffer; after insertion of the buffer, the switching response time was from 1.4 s in the bleaching state to 4.0 s in the coloring state, which was faster than the ECD without buffer (response time was from 1.5 s in the bleaching state to 5.2 s in the coloring state). In addition, we also survey the comparison of recent research on electrodes under various conditions in Table 4.

## 4. Conclusions

We established an appropriate electrode, Ti:V_2_O_5_, as a base to insert a buffer. According to our results, the buffer inserted on the Ti:V_2_O_5_ electrode film with exclusive surface morphology not only effectively increased the coloration efficiency (CE), but also promoted optical transmittance and durability. Moreover, strategies such as Ti:V_2_O_5_ films are based on a series of Ar/O_2_ gas flow ratios (1/2, 1/2.5, 1/3, and 1/3.5) on indium tin oxide (ITO) glass to establish the appropriate electrode.

Subsequently, we picked the Ti:V_2_O_5_ film with an Ar/O_2_ gas flow ratio of 1/3 as the best electrode base. This film had the largest cyclic voltammetry (CV) area to allow more Li ions into/out of the electrode interface and presented the highest optical transmittance (∆T) of 27%. Atomic force microscopy (AFM) was performed to obtain a deep understanding of the surface topography of the electrode interface. In addition, this value of Ti:V_2_O_5_ (Ar/O_2_ = 1/3) is helpful for optical transmittance, as it possesses the smallest average roughness (Ra = 3.53 nm) to reduce light scattering.

To distinguish the difference when the buffer was inserted on the electrode, we implemented many analyses. Regarding coloration efficiency (CE), the IrO_2_ buffer/Ti:V_2_O_5_ film achieved a higher value (96.1 cm^2^/C), providing large optical modulation, than the Ti:V_2_O_5_ film without buffer (33.9 cm^2^/C). Regarding crystallinity, the IrO_2_ buffer/Ti:V_2_O_5_ film, which has an intense diffraction peak at a 2θ angle of 34° and a preferential orientation of (101), is better than the Ti:V_2_O_5_ film, which has an amorphous structure. Regarding surface morphology, the IrO_2_ buffer/Ti:V_2_O_5_ film with a filamentary and interconnected structure, which provides larger inner pore and contact area, improves Li ion transfer in/out interface of the electrode compared with the Ti:V_2_O_5_ film with a featureless structure.

We successfully fabricated a buffered ECD via a vacuum cathodic arc plasma (CAP) technique. Our goal was to insert the IrO_2_ buffer to enhance the properties of the ECDs. Compared to the ECD without buffer, the buffered ECD promoted optical transmittance modulation (∆T = 57%) and maintained excellent durability of up to 98% (2% decay). This may provide a new insight into the development of energy devices. The superior electrochromic performance with the buffer layer makes our ECD a prominent candidate for use in smart windows with reduced energy demand.

## Figures and Tables

**Table 1:** Deposition parameters of IrO_2_ buffer on Ti:V_2_O_5_ counter electrode films.

No.	Film	Ar/O_2_ (Ar = 20 sccm)	W.P. (Torr)	DC Power (W)	Deposition Temp. (°C)	Deposition Time (s)	Thickness (nm)
Sample 1	Ti:V_2_O_5_	1/2	2.1 × 10^−3^	1200	100	80	80
Sample 2	Ti:V_2_O_5_	1/2.5	2.3 × 10^−3^	1200	100	80	80
Sample 3	Ti:V_2_O_5_	1/3	2.5 × 10^−3^	1200	100	80	80
Sample 4	Ti:V_2_O_5_	1/3.5	2.8 × 10^−3^	1200	100	80	80
Sample 5	IrO_2_ buffer	1/3	1.8 × 10^−3^	1250	100	10	20

**Table 2:** Deposition parameters of WO_3_ electrode film and ITO glass.

Target	Ar/O_2_ (sccm)	W.P. (Torr)	DC Power (W)	Deposition Time (min)	Deposition Rate (nm/min)	Deposition Temp. °C	Thickness (nm)
W Metal	1/3 (Ar = 100)	8 × 10^−3^	1500	15	13	50	200
ITO	1/3 (Ar = 100)	3 × 10^−3^	500	60	5	200	300

**Table 3:** Topographical parameters of Ti:V_2_O_5_ counter electrode films.

No.	Film	Ar/O_2_ Ratio	Image Surface Area (µm^2^)	Image Projected Surface Area (µm^2^)	Root Mean Square Roughness (nm)	Average Roughness (nm)
Sample 1	Ti:V_2_O_5_	1/2	1.33	1	7.22	5.63
Sample 2	Ti:V_2_O_5_	1/2.5	1.22	1	6.54	4.83
Sample 3	Ti:V_2_O_5_	1/3	1.10	1	5.01	3.91
Sample 4	Ti:V_2_O_5_	1/3.5	1.27	1	6.62	4.94

**Table 4:** Comparison of recent research on electrodes under various conditions.

Counter Electrode	Method	∆T (%)	CE (cm^2^/C)	Switching Time (t_c_/t_b_)	Ref.
IrO_2_ buffer/Ti:V_2_O_5_	CAP	57	96.1	4.0/1.4 s	This work
IrO_2_	CAP	50	-	4.8/1.5 s	[38]
V_2_O_5_	polyol-mediated synthesis	50	-	-	[8]
V_2_O_5_	polymer synthesis	33	89	20/23 s	[10]
Ti:V_2_O_5_	polyol synthesis	10	-	-	[11]
NiO	spray pyrolysis	37.4	42	8/10 s	[12]
V_2_O_5_	spin coating	31	-	8.2/6.3 s	[17]

## Data Availability

The data presented in this study are available on request from the corresponding author. The data are not publicly available due to privacy.
